# Theoretical Studies on Selectivity of HPK1/JAK1 Inhibitors by Molecular Dynamics Simulations and Free Energy Calculations

**DOI:** 10.3390/ijms24032649

**Published:** 2023-01-31

**Authors:** Huizhen Ge, Chunchao Tang, Yiting Pan, Xiaojun Yao

**Affiliations:** College of Chemistry and Chemical Engineering, Lanzhou University, Lanzhou 730000, China

**Keywords:** HPK1 inhibitor, selectivity mechanism, molecular dynamics simulation, binding free energy, umbrella sampling simulations

## Abstract

Hematopoietic progenitor kinase 1 (HPK1) is a negative regulator of T cell receptor, which has been regarded as a potential target for immunotherapy. Yu et al. observed the off-target effect of the high-throughput screening HPK1 kinase inhibitor hits on JAK1 kinase. The off-target effect is usually due to the lack of specificity of the drug, resulting in toxic side effects. Therefore, exploring the mechanisms to selectively inhibit HPK1 is critical for developing effective and safe inhibitors. In this study, two indazole compounds as HPK1 inhibitors with different selectivity towards JAK1 were used to investigate the selectivity mechanism using multiple computational methods, including conventional molecular dynamics simulations, binding free energy calculations and umbrella sampling simulations. The results indicate that the salt bridge between the inhibitor and residue Asp101 of HPK1 favors their selectivity towards HPK1 over JAK1. Information obtained from this study can be used to discover and design more potent and selective HPK1 inhibitors for immunotherapy.

## 1. Introduction

Hematopoietic progenitor kinase (HPK1), also known as MAP4K1, is a serine/threonine kinase that is a member of the mitogen-activated kinase kinase kinase kinase (MAP4K) family [1]. HPK1 is predomainantly expressed in hematopoietic cells and mainly regulates T cell activation through the TCR signaling pathway [2,3]. After TCR activation, HPK1 is phosphorylated on tryrosine 379 by kinases such as lymphocyte-specific protein tyrosine kinase (LCK), the activated HPK1 feedback phosphorylates Ser376 of Src homology 2 domain-containing leukocyte protein of 76 kDa (SLP76), which binds to negative regulator complex 14-3-3 [4,5,6,7]. This results in ubiquitination of lysine 30 on SLP76 and degration of SLP76 and subsequently limits strength and duration of TCR signals [8]. HPK1 kinase dead mice and HPK1 knockout mice models showed that loss of HPK1 kinase function enhanced T cell proliferation in response to TCR stimulation and reduced tumor growth and did not show fatal inflammation [3,9,10,11,12]. These studies have demonstrated that HPK1 inhibitors have potential applications in improving T cell function and combating immunosuppressive tumor microenvironments. Furthermore, the combination of HPK1 inhibitor and anti-programmed death-ligand-1 (anti-PD-L1) performed better anti-tumor immune effects than blocking individual targets [9,13,14]. Therefore, HPK1 inhibitors have potential roles in cancer immunotherapy.

Due to the structural similarities of the ATP binding site, kinase inhibitors typically display high promiscuity. Small inhibitors targeting specific kinase can be off-target in kinases of different families. Yu et al. observed off-target effects of the highpthroughput screening HPK1 kinase inhibitor hits on JAK1 kinase [15]. JAK1 is a member of the Janus kinase (JAK) family and plays a key role in cytokine-mediated inflammatory and autoimmune responses through the JAK/STAT signal pathway [16,17]. Off-target effect is usually due to the lack of specificity of the drug, resulting in toxic side effects. Therefore, designing potent HPK1 inhibitors with selectivity is very important. Yu et al. reported a series of indazole inhibitors as HPK1 inhibitors with different degrees of selectivity towards JAK1, such as XHS (751 fold selectivity over JAK1) and XHV (112 fold) [15]. The selective inhibitors may provide us with information to reveal the mechanism of the selectivity of ligand for HPK1 over JAK1.

**Figure 1:**
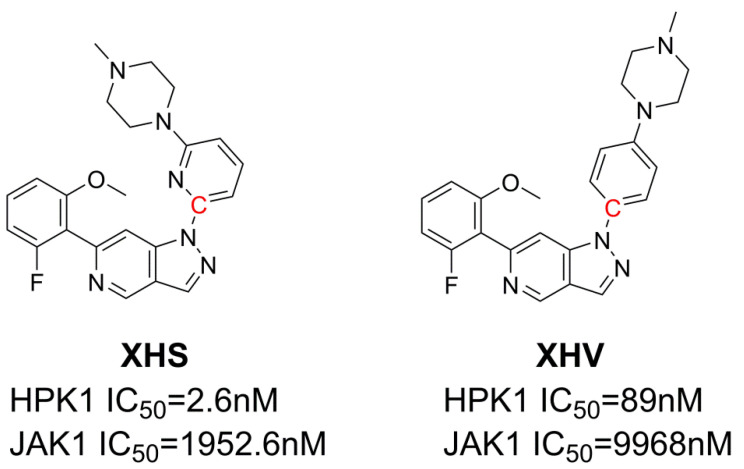
The chemical structures and IC_50_ of the inhibitors studied in this work.

Molecular dynamics simulations have emerged as a powerful approach to explore mechanisms of inhibitor selectivity [18,19,20,21,22,23,24,25,26,27,28,29,30,31]. In the present study, two indazole inhibitors, XHS and XHV, were selected to study their selective mechanisms towards HPK1 and JAK1. XHS and XHV are highly (751-fold; HPK1 IC_50_ = 2.6 nM, JAK1 IC_50_ = 1952.6 nM) and moderately (112-fold; HPK1 IC_50_ = 89 nM, JAK1 IC_50_ = 9968 nM) selective inhibitors, respectively, for HPK1 over JAK1. The chemical structures and IC_50_ of XHS and XHV are shown in Figure 1. We studied the selectivity of XHS and XHV by multiple molecular computational methods. MM-GBSA was used to determine the binding free energy. The umbrella sampling simulations were used to study the dissociation process of the two inhibitors from the HPK1/JAK1 binding pocket. The results demonstrated that the salt bridge interaction between the residue Asp101 of HPK1 and the inhibitor seems to be important for the selective inhibition of HPK1. Furthermore, the umbrella sampling results suggest that the dissociation process is important in kinase selectivity. Consequently, we suggest that, in addition to focusing on binding affinity, the dissociation process should also be taken into consideration.

## 2. Results and Discussion

### 2.1. Sequence Alignment of HPK1 and JAK1

**Figure 2:**
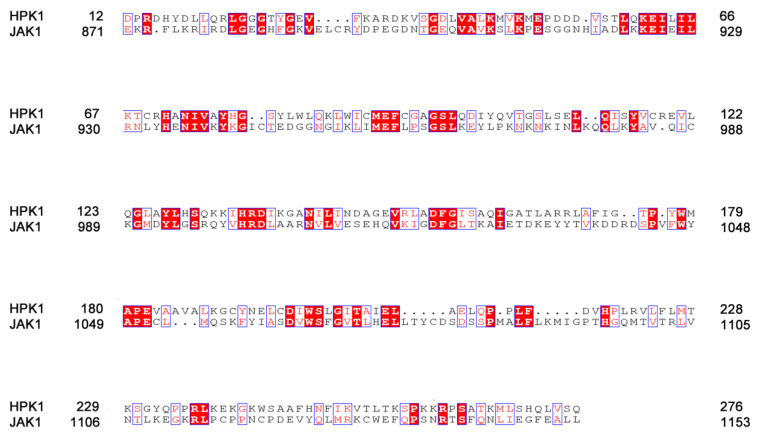
Sequence alignment of HPK1 and JAK1. The white characters with red background indicated that the amino acid sequences are completely identical, and the red characters with white background indicated that the amino acids are different but similar in properties.

To compare the difference between HPK1 and JAK1 from sequence, the ClustalW2 pairwise sequence alignment program tools for aligning two sequences in a pairwise manner, and the resulting sequence alignment was displayed by the online tool ESPript3.0 [32] in Figure 2. In it, the white characters with red background indicated that the amino acid sequences are completely identical, and the red characters with white background indicated that the amino acids are different but similar in properties. HPK1 shared 23.6% sequence identity and 53.5% sequence similarity with JAK1 in kinases domain and shared 46.5% sequence identity and 76.7% sequence similarity with JAK1 at ATP binding pocket.

### 2.2. The Conformational Stabilities of the Four Systems

**Figure 3:**
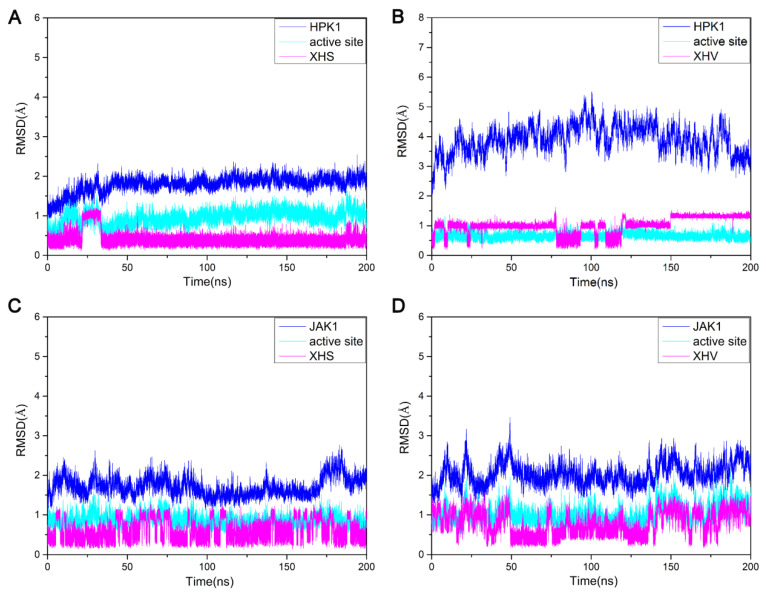
The RMSDs of four complexes. (**A**) HPK1-XHS complex, (**B**) HPK1-XHV complex, (**C**) JAK1-XHS complex, (**D**) JAK1-XHV complex.

A detailed analysis of root mean square deviation (RMSD) showed that the backbone atoms, active pocket atoms and ligand heavy atoms of the three systems HPK1-XHS, HPK1-XHV, JAK1-XHV tend to converge after 50 ns, indicating that the systems became sufficiently stable through 200 ns of simulations (Figure 3A,C,D). The protein backbone of the JAK1-XHS system is not as stable as the other three systems, but the active pocket atoms and ligand heavy atoms are stable throughout the simulations (Figure 3B). In general, through 200 ns conventional molecular dynamics simulations, we obtained more reasonable binding conformation for the two aligned systems JAK1-XHS and JAK1-XHV.

### 2.3. Binding Free Energy Calculations

To compare the binding affinities of two inhibitors towards HPK1 and JAK1, the binding free energy (ΔG_bind_), which includes gas-phase energy, solvation energy, and entropy term, was computed using MM-GBSA method. Table 1 summarized ΔG_bind_ for four systems and experimental binding free energies calculated from IC_50_ values. Results showed that the calculated ΔG_bind_ of XHS (−26.60 ± 7.08 kcal/mol) and XHV (−18.68 ± 6.92 kcal/mol) binding to HPK1, which corresponds to the order of experimental binding affinities (HPK1: XHS, IC_50_ = 2.6 nM; XHV, IC_50_ = 89 nM). The calculated ΔG_bind_ of inhibitors XHS (−18.09 ± 8.87 kcal/mol) and XHV (−10.48 ± 4.98 kcal/mol) binding to JAK1 are also in agreement with the experimental binding affinity order (JAK1: XHS, IC_50_ = 1952.6 nM; XHV, IC_50_ = 9968 nM). In addition, HPK1-selective inhibitor XHS has a lower calculated binding energy for HPK1 (ΔG_bind_ = −26.60 ± 7.08 kcal/mol) than JAK1 (ΔG_bind_ = −18.09 ± 8.87 kcal/mol) and shows a more significant difference in ΔG_bind_ values for HPK1 over JAK1 than XHV. In general, the difference in ΔG_bind_ reflects that XHS is a more potent selective HPK1 inhibitor over JAK1 than XHV.

To further study the driving force for selective binding of two inhibitors on HPK1 over JAK1, the binding enthalpy change (ΔH) was decomposed into independent binding free energy (Table 1) by MM-GBSA method. The results revealed that the primary driving force between inhibitors and kinases (HPK1 and JAK1) was the non-polar interaction energy, which is the sum of ΔE_vdW_ and ΔG_nonpl,sol_, while the polar interaction (the sum of ΔG_ele_ and ΔG_GB_) had a negative impact on binding. The ΔG_bind_ difference between inhibitor XHS and two kinases is polar interaction (9.52 ± 1.43 kcal/mol for HPK1 kinase, 17.47 ± 0.71 kcal/mol for JAK1 kinase). The ΔG_bind_ difference between inhibitor XHV and two kinases is non-polar interaction (−56.48 ± 2.90 kcal/mol for HPK1 kinase, −49.16 ± 3.99 kcal/mol for JAK1 kinase).

**Figure 4:**
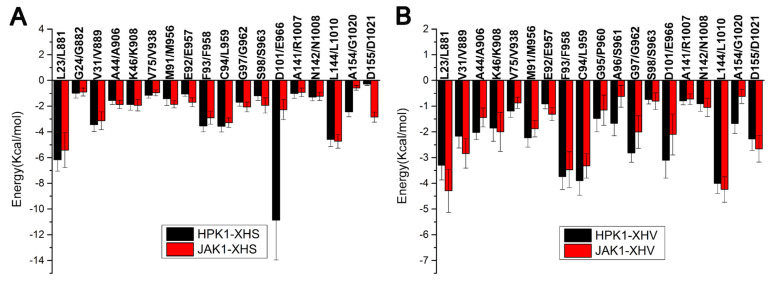
(**A**) Total energy contributions of inhibitor XHS with HPK1/JAK1, (**B**) total energy contributions of XHV with HPK1/JAK1.

In order to delve into the roles that interacting residues played in the binding of inhibitors to different kinases, the binding enthalpy change (ΔH) was decomposed to each residue. Figure 4 displays the residues with energy contributions higher than 0.5 kcal/mol. For inhibitor XHS, residues Leu23, Gly24, Val31, Ala44, Lys46, Val75, Met91, Glu92, Phe93, Cys94, Gly97, Ser98, Asp101, Ala141, Gln142, Leu144, Ala154 of HPK1 have favorable binding energy; Whereas, residues Leu881, Gly882, Val889, Ala906, Lys908, Val938, Met956, Glu957, Phe958, Leu959, Gly962, Ser963, Glu966, Arg1007, Gln1008, Leu1010, Gly1020, Asp1021 of JAK1 contribute favorably to the inhibitor XHS binding. Among these residues, the interaction of inhibitor XHS with the residue Asp101 of HPK1 shows significant difference with the residue Glu966 of JAK1 (Figure 4A). Besides that, the difference in the binding free energy of HPK1/Ala154 and JAK1/Gly1020 with XHS plays a role in selectivity. For inhibitor XHV, residues Leu23, Val31, Ala44, Lys46, Val75, Met91, Glu92, Phe93, Cys94, Gly95, Ala96, Gly97, Ser98, Asp101, Ala141, Gln142, Leu144, Ala154, Asp155 of HPK1 have favorable binding energy; the residues Leu881, Val889, Ala906, Lys908, Val938, Met956, Glu957, Phe958, Leu959, Pro960, Ser961, Gly962, Ser963, Glu966, Arg1007, Gln1008, Leu1010, Gly1020, Asp1021 of JAK1 favorably influence the inhibitor XHV’s binding (Figure 4B). The differences of binding free energy of HPK1/Ala96, Gly97, Asp101, Ala154 and JAK1/Ser961, Gly962, Glu966, Gly1020 with XHV are responsible for the selectivity.

### 2.4. Hydrogen Bond and Salt Bridge Analysis

Hydrogen bond (H-bond) occupancy was calculated to track the formation of hydrogen bonds between the inhibitors and their corresponding targets during the MD simulations. According to Table 2, in HPK1-XHS system, the stable H-bond is formed between backbone NH of Cys94 and the N atom of indazole scaffold (90.80% occupancy). In JAK1-XHS system, the stable H-bond is formed between backbone NH of Leu959 and the N atom of indazole scaffold (86.64% occupancy). In HPK1-XHV system, the stable H-bond is formed between backbone NH of Cys94 and the N atom of indazole scaffold (82.36% occupancy). In JAK1-XHV system, the stable H-bond is formed between backbone NH of Leu959 and the N atom of indazole scaffold (65.76% occupancy). The H-bond occupancy of HPK1 systems is higher than that of JAK1 systems, which can explain the fact that XHS and XHV have better inhibition activity for HPK1 over JAK1. The salt bridge is double-charged hydrogen bond based on the classification of hydrogen bond proposed by Gilli et al. [33,34,35,36]. Next the salt bridge occupancy was calculated to monitor the formation of salt bridge between the inhibitors and their corresponding targets. As shown in Table 3, salt bridges were detected in only two systems, and the salt bridge between the protonated NH+ of piperazine and the side chain carboxyl of Asp101 (OD with 62.03% occupancy) in the HPK1-XHS system is more stable than the salt bridge between protonated NH+ of piperazine with the side chain carboxyl of Glu966 (OE with 7.41% occupancy) in the JAK1-XHS system. In general, the salt bridge between inhibitor and Asp101 is critical for enhanced potency and selectivity.

### 2.5. The Binding Mode between Kinases (HPK1/JAK1) and Inhibitors (XHS/XHV)

**Figure 5:**
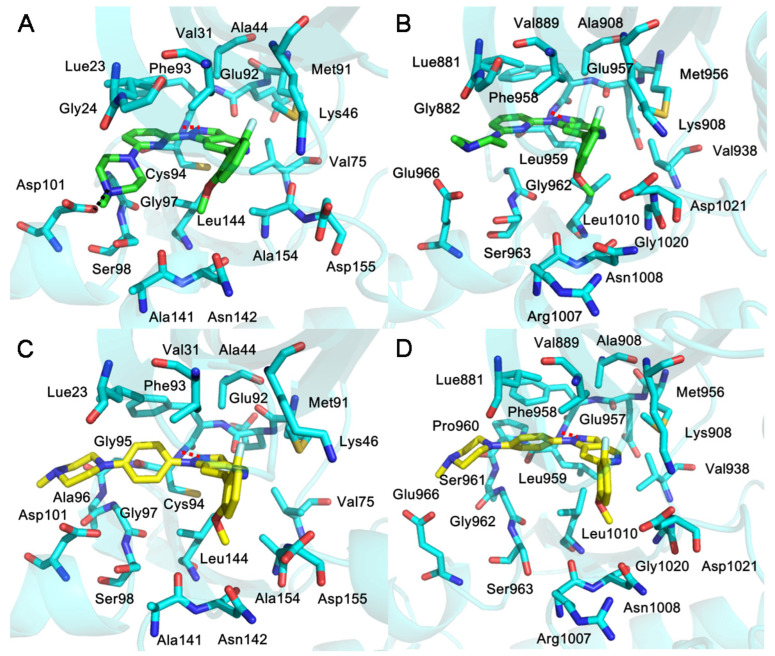
The binding mode of (**A**) HPK1-XHS (**B**) JAK1-XHS (**C**) HPK1-XHV (**D**) JAK1-XHV systems. The inhibitor XHS is shown as green sticks, the inhibitor XHV is shown as yellow sticks and the key residues of HPK1 and JAK1 kinase binding to inhibitors are shown as cyan sticks. Hydrogen bonds and salt bridges kinases and inhibitors are indicated by red and black dashes, respectively.

In order to study the detailed binding mode between the two inhibitors (XHS/XHV) and the two kinases (HPK1/JAK1) and explain the difference in binding affinity, the last frame of the four system trajectories were extracted. There are similar binding pockets between HPK1 and JAK1. The binding modes of inhibitors with kinases (HPK1 and JAK1) are depicted in Figure 5. According to Figure 5A, the structure of XHS bound to HPK1 demonstrated (1) that the residues Val31, Lys46, Ala141, Asn142 and Ala154 surround 2-fluoro-6-methoxyphenyl group and more lipophilic fluorine group point to P-loop of protein; (2) that the residues Ala44, Val75, Met91, Glu92, Phe93, Cys94 and Leu144 surround indazole core, and the backbone NH of Cys94 forms a H-bond with the N of the indazole; (3) that the residues Leu23, Gly24, Gly97, Ser98 and Asp101 surround meta piperazine substituted pyridine group, and salt bridge is formed between Asp101 and protonated NH+ on piperazine. According to Figure 5B, the structure of XHS bound to JAK1 demonstrated (1) that the residues Val859, Lys908, Arg1007, Asn1008, Gly1020 and Asp1021 surround 2-fluoro-6-methoxyphenyl group and the more lipophilic fluorine group point to P-loop of protein; (2) that the residues Ala906, Val938, Met956, Glu957, Phe958, Leu959 and Leu1010 surround indazole core and the backbone NH of Leu959 forms a H-bond with the N of the indazole core; and (3) that the residues Leu881, Gly882, Gly962, Ser963 and Glu966 surround meta piperazine substituted pyridine group and an unstable salt bridge is formed between Glu966 and protonated NH+ on piperazine. As depicted in Figure 5C, the structure of XHV bound to HPK1 demonstrated that (1) the residues Val31, Lys46, Ala141, Asn142, Ala154 and Asp155 surround 2-fluoro-6-methoxyphenyl group, the more lipophilic fluorine group point to P-loop of protein; (2) that the residues Ala44, Val75, Met91, Glu92, Phe93, Cys94 and Leu144 surround indazole core and the backbone NH of Cys94 forms a H-bond with the N of the indazole; and (3) that the residues Leu23, Ala96, Gly97, Ser98 and Asp101 surround para piperazine substituted phenyl group, which is located at the entrance of the binding site. As depicted in Figure 5D, the structure of XHV bound to JAK1 demonstrated (1) that the residues Val889, Lys908, Arg1007, Asn1008, Gly1020 and Asp1021 surround 2-fluoro-6-methoxyphenyl group and the more lipophilic fluorine group point to P-loop of protein; (2) that the residues Ala906, Val938, Met956, Glu957, Phe958, Leu959 and Leu1010 surround indazole core and the backbone NH of Leu959 forms a H-bond with the N of the indazole core; and (3) that the residues Leu881, Ser961, Gly962, Ser963 and Glu966 surround para piperazine substituted phenyl group, which is located at the entrance of the binding site. According to the results of our simulation, there are similar binding sites between HPK1 and JAK1. The comparative results of the binding sites suggest that the salt-bridge interaction between Asp101 of HPK1 kinase and the inhibitor plays a dominant role in the binding of HPK1 selective inhibitors.

### 2.6. Unbinding Pathways of Inhibitors (XHS and XHV) Dissociating from Kinases (HPK1 and JAK1)

**Figure 6:**
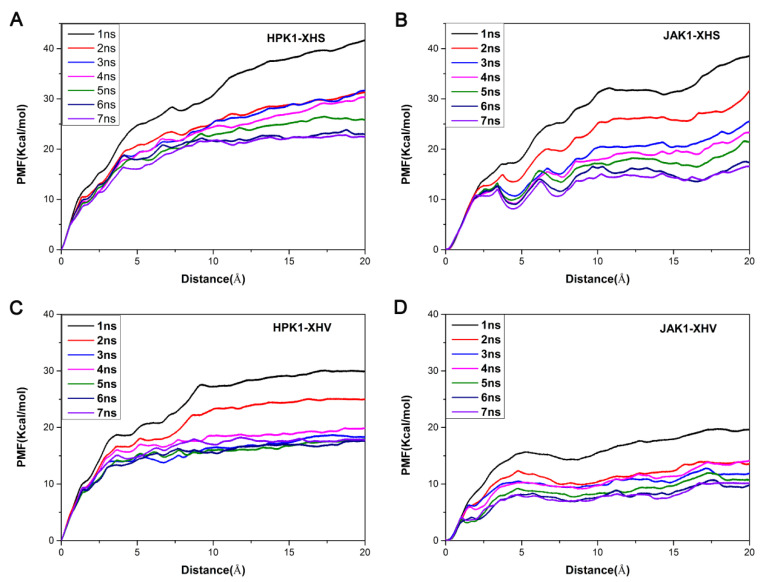
Convergence of the PMFs calculated for four systems by umbrella sampling simulations. (**A**) HPK1-XHS system, (**B**) JAK1-XHS system, (**C**) HPK1-XHV system, (**D**) JAK1-XHV system.

**Figure 7:**
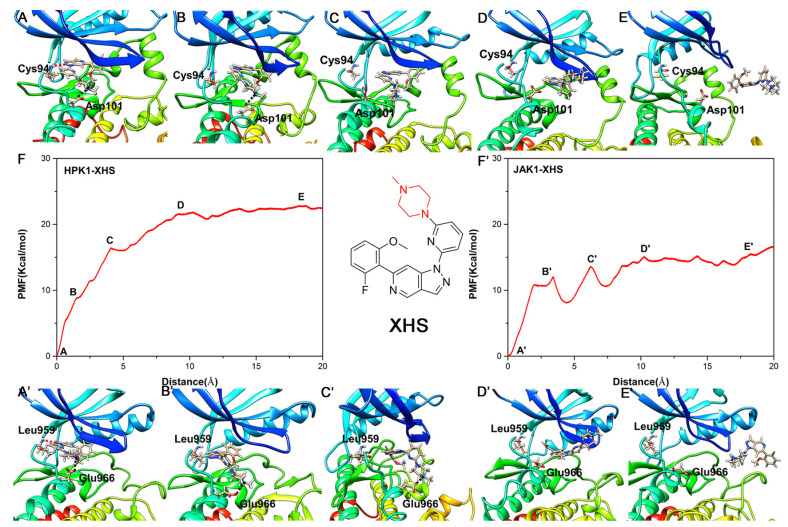
The unbinding process of inhibitor XHS dissociating from binding pocket of HPK1 kinase (**A**–**E**) and the corresponding PMF curve (**F**), the unbinding process of inhibitor XHS dissociating from binding pocket of JAK1 kinase (**A**’–**E**’) and the corresponding PMF curve (**F**’).

**Figure 8:**
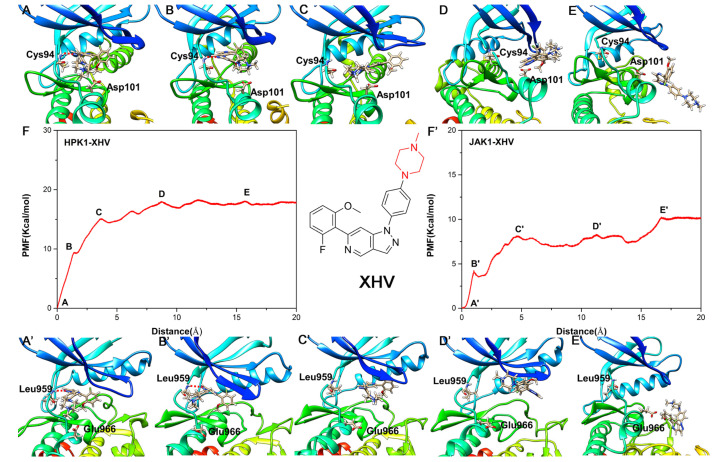
The unbinding process of inhibitor XHV dissociating from binding pocket of HPK1 kinase (**A**–**E**) and the corresponding PMF curve (**F**), the unbinding process of inhibitor XHV dissociating from binding pocket of JAK1 kinase (**A**’–**E**’) and the corresponding PMF curve (**F**’).

In this study, the last frame of the four system trajectories were used as the initial structures for the umbrella sampling simulations. As depicted in Figure 6, each system reaches convergence of PMFs after performing 7 ns umbrella sampling simulations. As depicted in Figure 7 and Figure 8, the PMF depth derived from the umbrella sampling simulations of the HPK1 system is higher than that of the JAK1 system. To further determine the detailed process of dissociation, the last 1 ns in each system was used for analysis. As indicated in Figure 7F, minimum value of PMF (0 kcal/mol) was calibrated to 0 Å on RC to indicate the binding state of XHS in HPK1/JAK1. The RCs were extended from 0 to 20 Å represent XHS 20 Å away from the RCs. XHS dissociated from RC with 22.43 ± 0.07 kcal/mol for HPK1 and 15.09 ± 0.86 kcal/mol for JAK1 (Table 4); the difference is 7.34 kcal/mol. This finding suggested that the inhibitor XHS dissociated from JAK1 much easier than that of XHS from HPK1 during the dissociation process. When XHS escapes from the binding pocket of HPK1 at point B of the PMF curve (~1.6 Å, Figure 7F), an obvious energy barrier is first displayed, where the 2-fluoro-6-methoxyphenyl group moves outward along the RC direction and the H-bond between the N of the indazole core and the NH of Cys94 is broken. Afterward, the piperazine moiety of XHS flips outward and the salt bridge with Asp101 is broken (ascending region B-C in Figure 7F). Next, the meta piperazine substituted pyridine group is exposed to the solvent (point D in Figure 7F). Finally, XHS completely dissociated from HPK1 (point E in Figure 7F). For the JAK1-XHS system, the PMF curve rises significantly in region A’-B’ (Figure 7F’), where the 2-fluoro-6-methoxyphenyl group moves outward along the RC direction the H-bond between the N of indazole core and the NH of Leu959 is broken. Then the meta piperazine substituted pyridine group flipped and exposed to solvent (point C’, Figure 7F’), followed by the cleavage of the weak salt bridge between the piperazine moiety of XHS with Glu966 of JAK1 (point D’, Figure 7F’). After that, the inhibitor XHS is completely dissociated from the binding pocket of JAK1 (point E’ in Figure 7F’). In general, the bioselectivity of the inhibitor XHS for HPK1 over JAK1 can be explained by stable salt bridge between the piperazine moiety of XHS and residue Asp101 of HPK1.

The PMF curve of inhibitor XHV exhibits a completely different behavior compared to the PMF curve of inhibitor XHS. According to Table 4, XHV dissociated from RC with 17.68 ± 0.16 kcal/mol for HPK1 and 10.02 ± 0.29 kcal/mol for JAK1; the difference is 7.66 kcal/mol. For the HPK1-XHV system, the PMF curve first rises significantly (regions A-B-C, Figure 8F); the piperazine moiety of the inhibitor XHV is flipped, and the H-bond between the N of indazole core and the NH of Cys94 is broken. Then the piperazine moiety is released from the binding pocket, and the inhibitor XHV eventually dissociated from HPK1 (regions D-E in Figure 8F). Distinct from that of HPK1, two stages of sharp rise in PMF (regions A’-B’-C’ and D’-E’ in Figure 8F’) are observed before the release of the inhibitor XHV from the binding pocket of JAK1. With the increase in PMF, the 2-fluoro-6-methoxyphenyl group of inhibitor XHV moves to entrance of the binding pocket, and then the H-bond between indazole core and Leu959 is broken.(regions A’-B’-C’ in Figure 8F’). The indazole core is then flipped and exposed to the solvent, and then the inhibitor XHV is completely dissociated from the binding pocket of JAK1 (region D’-E’ in Figure 8F’). The difference in PMF during dissociation of inhibitor XHV from HPK1 and JAK1 is due to the difference in the hydrophobic interaction of the 2-fluoro-6-methoxyphenyl group and piperazine moiety of XHV with kinases. In summary, the dynamic and energetic information obtained in this research is helpful for understanding binding/unbinding mechanism of the inhibitors XHS/XHV to and from HPK1/JAK1.

## 3. Materials and Methods

### 3.1. Sequence Alignment and Structure Superposition

The crystal structure of human HPK1 kinase with each of ligands XHS and XHV (PDB ID: 7L25 and 7L24) [15] and JAK1 kinase bound with inhibitor (PDB ID: 6SMB) [37] were downloaded from the Protein Data Bank (https://www.rcsb.org/, accessed on 12 April 2021). The ClustalW2 [38] pairwise sequence alignment tools were used to align the human HPK1 (PDB: 7L25) and JAK1 (PDB: 6SMB) in FASTA format to determine the proportion of conserved and unique residues. To construct complex structures of 6SMB and inhibitors XHS and XHV, models 7L25, 7L24 and 6SMB were structurally aligned using PyMOL Molecular Graphics System, and the inhibitors XHS and XHV were merged into the binding pocket of JAK1. The crystal structure of HPK1-XHS, HPK1-XHV and aligned structure JAK1-XHS, JAK1-XHV were used as the initial structure for the following calculations.

### 3.2. Molecular Dynamics Simulation

The complexes structure of HPK1-XHS (crystal structure), JAK1-XHS, HPK1-XHV (crystal structure) and JAK1-XHV were used as starting coordinates for the MD simulations. The Amber18 package [39] was used to perform all of the MD simulations. The restrained electrostatic potential (RESP), calculated by Hartree–Fork (HF) method with 6-31G* basis set in the Gaussian09 [40], was used to fit the partial charges for the inhibitor XHS and XHV. The kinase parameters were described using the ff14SB force field, and the inhibitors were parameterized using the general AMBER force field (gaff). TIP3PBOX water molecules were used to solvate the complex 12 Å away to the boundary. The systems were minimized, heated and equilibrated after neutralizing the systems with chloride ions and sodium ions. At 300 K and 1.0 atmosphere pressure, 200 ns MD simulations were performed in NPT ensemble. The trajectory analysis was carried out using AMBER18′s cpptraj module.

### 3.3. Binding Free Energy Calculations

A total of 1000 snapshots extracted from each system’s last 50 ns trajectories were used to calculate binding free energy using the MM-GBSA [41,42,43] method. The calculation for the binding free energy is as follows.

(1) $$\Delta G_{\mathrm{bind}}=G_{\mathrm{complex}}-\left(G_{\mathrm{receptor}}+G_{\mathrm{ligand}}\right)$$

(2) $$G_{\mathrm{bind}}=\Delta H-T\Delta S=E_{\mathrm{gas}}+G_{\mathrm{sol}}-T\Delta S$$

(3) $$E_{\mathrm{gas}}=E_{\mathrm{int}}+E_{\mathrm{ele}}+E_{\mathrm{vdW}}$$

(4) $$G_{\mathrm{sol}}=G_{\mathrm{GB}}+G_{\mathrm{nonpl},\mathrm{sol}}$$

(5) $$G_{\mathrm{nonpl},\mathrm{sol}}=\gamma *\mathrm{SASA}$$

In Equation (1), G_complex_, G_receptor_, G_ligand_ represent the free energy of complex, receptor and ligand molecules, respectively. In Equation (2), E_gas_ and G_sol_ represent the gas-phase energy and the solvation free energy. TΔS represents the change of conformational entropy when the ligand binds at temperature T. In Equation (3), E_gas_ consists of internal energy (E_int_), electrostatic energy (E_ele_) and van der Waals energy (E_vdW_). In Equation (4), G_sol_ consists of polar solvation energy (G_GB_) and nonpolar solvation energy (G_nonpl_,_sol_). In Equation (5), G_nonpl_,_sol_ was calculated by the SASA using a water probe radius of 1.4 Å. The surface tension constant γ was determined to be 0.0072 kcal/(mol·Å2) [44]. Due to the long computational time, the entropy term (−TΔS) was calculated using only 50 of the 1000 snapshots.

### 3.4. Free Energy Decomposition Analysis

In order to gain detailed information of the protein–ligand binding, MM-GBSA was utilized to decompose the interaction energies to each residue without consideration the contribution of entropy term. The essential residues of the indazole ihibitors for HPK1 over JAK1 binding are identified by the individual residue contributions derived by MM-GBSA energy decomposition, which also help us design more selective HPK1 inhibitors.

### 3.5. Umbrella Sampling Simulations

To characterize the unbinding process of each inhibitor from its corresponding targets, umbrella sampling simulations [45] were carried out to elucidate the drug binding selectivity using the pmemd program in AMBER18. The last frame of the four system trajectories were used as the initial structures for the umbrella sampling simulations. The distance between the carbon atom of inhibitors (red atom in Figure 1) and CA atom of Phe at hinge (HPK1 is Phe93, JAK1 is Phe958) was selected as reaction coordinates (RCs) using the tunnel prediction tool CAVER3.0 [46,47,48,49]. The RC was divided into 41 windows with a step of 0.5 Å. To guarantee convergence for each system, 7 ns MD simulations were run for each window. The residues at the end of each system are subjected to a force constant of 5 kcal mol^−1^ Å^−2^ to avoid the drift of receptor–ligand complexes. The umbrella sampling force constant was set to 5 kcal mol^−1^ Å^−2^. The weighted histogram analysis method (WHAM) [50,51] was used to determine the potential of mean force (PMF) values.

## 4. Conclusions

In order to investigate the selective inhibition mechanism for HPK1 inhibitors over JAK1, two indazole inhibitors’ binding selectivity was clarified using a computational approach that include all-atom conventional MD simulation, MM-GBSA free energy calculation and umbrella sampling simulations. The difference in HPK1 inhibitor activity between the two inhibitors is mainly due to the fact that the para-piperazine in ligand XHV extends to the solvent front, while the piperazine in ligand XHS changes direction to interact with Asp101, and the binding preference of the inhibitor XHS to HPK1 over JAK1 is controlled by the salt-bridge interaction of the piperazine ring with the carboxyl group on Asp101. The results of this study will offer useful recommendations for the rational development of HPK1 inhibitors with better selectivity over JAK1.

## Figures and Tables

**Table 1:** Binding free energies for four systems and experimental binding free energies calculated from IC_50_ values of two inhibitors on HPK1 and JAK1.

Energy	HPK1-XHS	JAK1-XHS	HPK1-XHV	JAK1-XHV
ΔE_ele_	−31.58 ± 13.41	−53.77 ± 19.26	−37.81 ± 9.73	−41.81 ± 19.85
ΔE_vdw_	−49.88 ± 2.80	−48.37 ± 2.91	−50.30 ± 2.67	−43.62 ± 3.57
ΔE_gas_	−81.45 ± 14.14	−102.14 ± 19.47	−88.11 ± 9.85	−85.43 ± 20.40
ΔG_GB_	41.10 ± 11.98	71.24 ± 18.55	55.18 ± 9.55	57.67 ± 19.46
ΔG_nonpl,sol_	−5.99 ± 0.25	−5.99 ± 0.29	−6.18 ± 0.23	−5.54 ± 0.42
ΔG_sol_	35.11 ± 11.88	65.25 ± 18.42	49.00 ± 9.54	52.14 ± 52.14
ΔG_pl_	9.52 ± 1.43	17.47 ± 0.71	17.37 ± 0.18	15.86 ± 0.39
ΔG_nonpl_	−55.87 ± 3.05	−54.36 ± 3.20	−56.48 ± 2.90	−49.16 ± 3.99
ΔH (GB)	−46.35 ± 3.91	−36.89 ± 3.19	−39.11 ± 2.89	−33.30 ± 3.78
−TΔS	19.75 ± 5.85	18.80 ± 8.28	20.43 ± 6.32	22.82 ± 3.25
ΔG	−26.6 ± 7.08	−18.09 ± 8.87	−18.68 ± 6.92	−10.48 ± 4.98
IC_50_	2.6 nM	1952.6 nM	89 nM	9968 nM
ΔGexp ^a^	−11.71	−7.79	−9.61	−6.82

^a^$\Delta \mathrm{Gexp}\approx -{\mathrm{RTlnIC}}_{50}$.

**Table 2:** Hydrogen bond interaction between the inhibitors and kinases HPK1/JAK1.

Acceptor	Donor	Occupancy (%)	Distance (Å)	Angle (°)
HPK1-XHS				
XHS@N5	Cys94@HN	90.80	3.17	160.46
JAK1-XHS				
XHS@N5	Leu959@HN	86.64	3.20	150.60
HPK1-XHV				
XHV@N4	Cys94@HN	82.36	3.14	152.55
JAK1-XHV				
XHV@N4	Leu959@HN	65.76	3.25	153.23

**Table 3:** Salt bridge interaction between the inhibitors and kinases HPK1/JAK1.

System	Positively Charged Residue	Negatively Charged Residue	Occupancy (%)	Diatance (Å)
HPK1-XHS	XHS@N1	Asp101@OD	62.03	2.86
JAK1-XHS	XHS@N1	Glu966@OE	7.41	2.86

**Table 4:** PMFs of the inhibitor unbinding from the ATP pocket based on the US simulations.

Energy	HPK1-XHS	JAK1-XHS	HPK1-XHV	JAK1-XHV
PMF/kcal∙mol^−1^	22.43 ± 0.07	15.09 ± 0.86	17.68 ± 0.16	10.02 ± 0.29
IC_50_/nM	2.6	1952.6	89	9968
ΔGexp/kcal∙mol^−1^	−11.71	−7.79	−9.61	−6.82

## Data Availability

Not applicable.
